# EHPnet: NREL Biomass Research

**Published:** 2008-06

**Authors:** Erin E. Dooley

http://www.nrel.gov/biomass/

The Department of Energy National Renewable Energy Laboratory (NREL) conducts numerous research, development, and demonstration projects in the field of biomass-based energy. Easy-to-understand, up-to-date information about these efforts is available in the “Biomass Research” section of the NREL website.

Under “Biomass Basics,” visitors find a glossary of biomass terms and a consumer guide, *Exploring Ways to Use Biomass Energy*. A short video featuring NREL scientists explains how cellulosic biomass such as corn waste and switch-grass is converted to liquid fuels as well as the environmental and economic benefits of these fuels. A FAQ section is organized into eight broad topics including biochemical conversion, biodiesel, ethanol, and feedstocks.

More technical materials are available in the “Energy Analysis & Tools” section, which includes three main focal points: technoeconomic analysis, life cycle assessments, and tools. Under “Publications,” visitors can access the Biomass Document Database, which archives public documents dating all the way back to 1980, or they may download project fact sheets, posters, and other publications.

